# An overview on pharmaceutical applications of phosphodiesterase enzyme 5 (PDE5) inhibitors

**DOI:** 10.1007/s11030-024-11016-2

**Published:** 2024-11-27

**Authors:** Mohamed T. M. Nemr, Mostafa A. Abdelaziz, Mohamed Teleb, Ahmed E. Elmasry, Yaseen A. A. M. Elshaier

**Affiliations:** 1 Pharmaceutical Organic Chemistry Department, Faculty of Pharmacy, Cairo University, Kasr El-Eini Street 11562, Cairo, Egypt; 2 Department of Chemistry, College of Wooster, Wooster, OH 44691 USA; 3 Department of Pharmaceutical Chemistry, Faculty of Pharmacy, Alexandria University, Alexandria, 21521 Egypt; 4 Organic & Medicinal Chemistry Department, Faculty of Pharmacy, University of Sadat City, Menoufia, Egypt; 5 Faculty of Pharmacy, Alamein International University (AIU), Alamein City, Alamein City, 5060310 Egypt

**Keywords:** PDE5 enzymes, Erectile dysfunction, Type 2 diabetes, Antiproliferative, Anti-Alzheimer and anti-inflammatory

## Abstract

Phosphodiesterase enzyme 5 (PDE5) inhibitors have emerged as one of the leading molecules for the treatment of erectile dysfunction (ED). PDE5 inhibitors are categorized structurally into several classes. PDE5 inhibitors have been a multidisciplinary endeavor that attracts the attention of researchers because of their multiple pharmaceutical applications. Beyond their action on ED, PDE5 inhibitors are widely used in treatment of benign prostatic hypertrophy (BPH), Eisenmenger’s syndrome, Raynaud’s Disease, Intrauterine growth retardation (IUGR), Mountain sickness, Bladder pain syndrome/interstitial cystitis (BPS/IC), pulmonary arterial hypertension and type II diabetes (insulin resistance). In addition, PDE5 inhibitors also show promising antiproliferative activity, anti-Alzheimer and COX-1/COX-2 inhibitory activity (anti-inflammatory). Pharmacokinetics, Pharmacogenetics and toxicity of PDE5 inhibitors were finally explored. The diverse therapeutic applications, the high feasibility of structural modification and the appropriate pharmacokinetic properties of PDE5 inhibitors have motivated researchers to develop new scaffolds that have been either under clinical trials or approved by FDA and utilize them to overcome some recent global concerns, such as COVID-19.

## Introduction

Phosphodiesterases (PDEs) are a family of 11 enzymes that exist in almost all mamalian cells [1]. PDEs deactivate intracellular signal transduction mediators, such as cyclic adenosine monophosphate (cAMP) and cyclic guanosine monophosphate (cGMP) by hydrolyzing 3′, 5′-cyclic phosphate moiety into inactive corresponding 5′-nucleotide [2]. cAMP and cGMP are involved in the transmission of diverse physiological stimuli and regulation of numerous physiological processes, such as vascular resistance, cardiac output, visceral motility, immunological response [3], inflammation [4], neuroplasticity, vision [5], and reproduction [6]. Moreover, the ubiquitously present PDEs are crucial for regulation of intracellular levels of cAMP and cGMP [7]. Furthermore, inhibiting PDEs impedes the metabolism of cAMP and cGMP, leading to an increase in their levels with subsequent prolonged biological effects that depend on the type of cell involved [8]. In this review, The PDE superfamily will be discussed briefly and highlighted more on the PDE5 and its properties, as well as the pharmaceutical applications of PDE5 inhibitors, including some Food and Drug Administration (FDA)-approved drugs and other analogs that have been recently developed by researchers.

## Phosphodiesterase superfamily

The PDEs superfamily contains 11 different gene families: PDE1, PDE2, PDE3, PDE4, PDE5, PDE6, PDE7, PDE8, PDE9, PDE10 and PDE11. The PDEs differ from each other in terms of localization or tissue distribution, regulation pattern, and the specificity of their inhibitors [9]. PDEs are widely present in several intracellular organelles, such as cytosol, plasma membranes, endoplasmic reticulum, nuclear membranes, and cytoskeleton [10, 11]. Only PDE4, PDE7, and PDE8 can hydrolyze cAMP, while cGMP is only hydrolyzed by PDE5, PDE6, and PDE9. PDE1 and PDE2 take both nucleotides as a substrate [9, 12]. The PDE family members with their substrates, some exmples of their inhibitors, in addition to their therapeutic applications are summerized in Table 1 [13–16].

**Table 1:** Phosphodiesterase superfamily

PDE family	Substrate	Inhibitors	Therapeutic applications
PDE1	cAMP/cGMP	Vinpocetine, Nicardipine, Lenrispodun [13]	Dementia, memory loss Functional magnetic resonance imaging (fMRI) [13] urge incontinence and low compliance bladder [14],
PDE2	cAMP/cGMP	EHNA, BAY-60–7550	Sepsis Acute respiratory Distress syndrome Memory loss Antiplatelets [15], heart failure and cardiac arrhythmias, atherosclerosis, [16]
PDE3	cAMP/cGMP	pimobendan, anagrelide, milrinone, cilostazol, amrinone, vesnarinone	Ischemic and idiopathic dilated cardiomyopathy [17]
PDE4	cAMP	Rolipram Denbufylline, Cilomilast Roflumilast	Asthma, glomerulonephritis, and COPDs Bipolar disorder, autoimmune disease Brain infection and organ transplantation [18]
PDE5	cGMP	Sildenafil (Viagra) Zaprinast, Dipyridamole Ariflo, Vardenafil, Tadalafil	Chronic renal failure cardiovascular disease, erectile dysfunction, Transplantation of organs [19]
PDE6	cGMP	Zaprinast, Dipyridamole Vardenafil, Tadalafil	Retinal diseases [20]
PDE7	cAMP	Dipyridamole, Thiadiazole	Disorders of the immune system and airways [21]
PDE8	cAMP	Dipyridamole	Applications in immunology [22]
PDE9	cGMP	Zaprinast	Hypoglycemic effects [23]
PDE10	cAMP, cGMP	Theophylline, Caffeine, Papaverine	Psychiatric and neurodegenrative diseases [24]
PDE11	cAMP, cGMP	Tadalafil	Antitumor, Anti-inflammatory [25]
Dual substrate PDEs	cAMP/cGMP	Lixazinone, Cilostamide Milrinone, Cilostazol Dihydropyridazinone Dipyridamole, Papaverine, Tadalafil, Zaprinast, Dipyridamole	Glomerulonephritis Congestive heart failure ThrombosisPulmonary hypertension Treatsment of Schizophrenia, Proposed enhancements to human testicular performance [19–23]

## Properties of PDE5

**Fig. 1:**
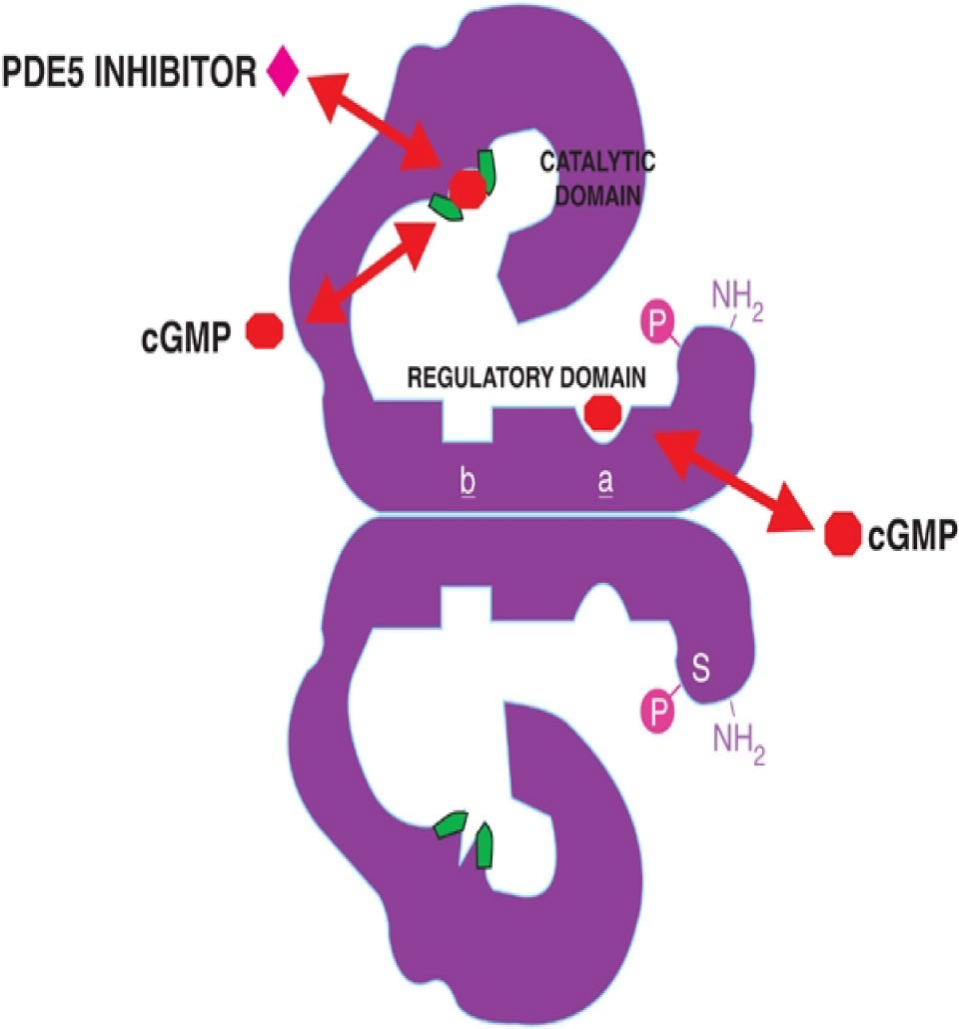
The PDE5 structure with its domains and binding sites

PDE5 is a dimer protein; each monomer is composed of two domains (Fig. 1): a catalytic domain that catalyzes the breakdown of cGMP to 5-GMP, and a regulatory domain. The regulatory domain contains allosteric GMP binding sites (non-catalytic sites, a and b), to which binding of cGMP increases the affinity of the catalytic site to cGMP and hence increases the rate of cGMP hydrolysis. It also contains a phosphorylation site (P) at serine 92. When PDE5 gets phosphorylated by protein kinase G (PKG), the enzymatic activity is augmented, and thus the affinity of PDE5 allosteric sites to cGMP and the catalytic activity increase.

Those effects suggest that PDE5 is critically involved in negative feedback regulation of cellular cGMP.

As shown in Fig. 1, the two allosteric cGMP-binding sites, the phosphorylation site (Serine 92), and the regulatory domain are all located in the amino-terminal region of PDE5. The two Zn^2+^ binding motifs and a substrate site for cGMP binding are found in the catalytic domain of the carboxyl-terminal region of the protein. Amino acids that are potentially involved in the catalytic mechanism are indicated in green and those involved in cGMP binding in an allosteric or catalytic site are indicated in red [26–28].

## Mechanism of action of PDE5 inhibitors

**Fig. 2:**
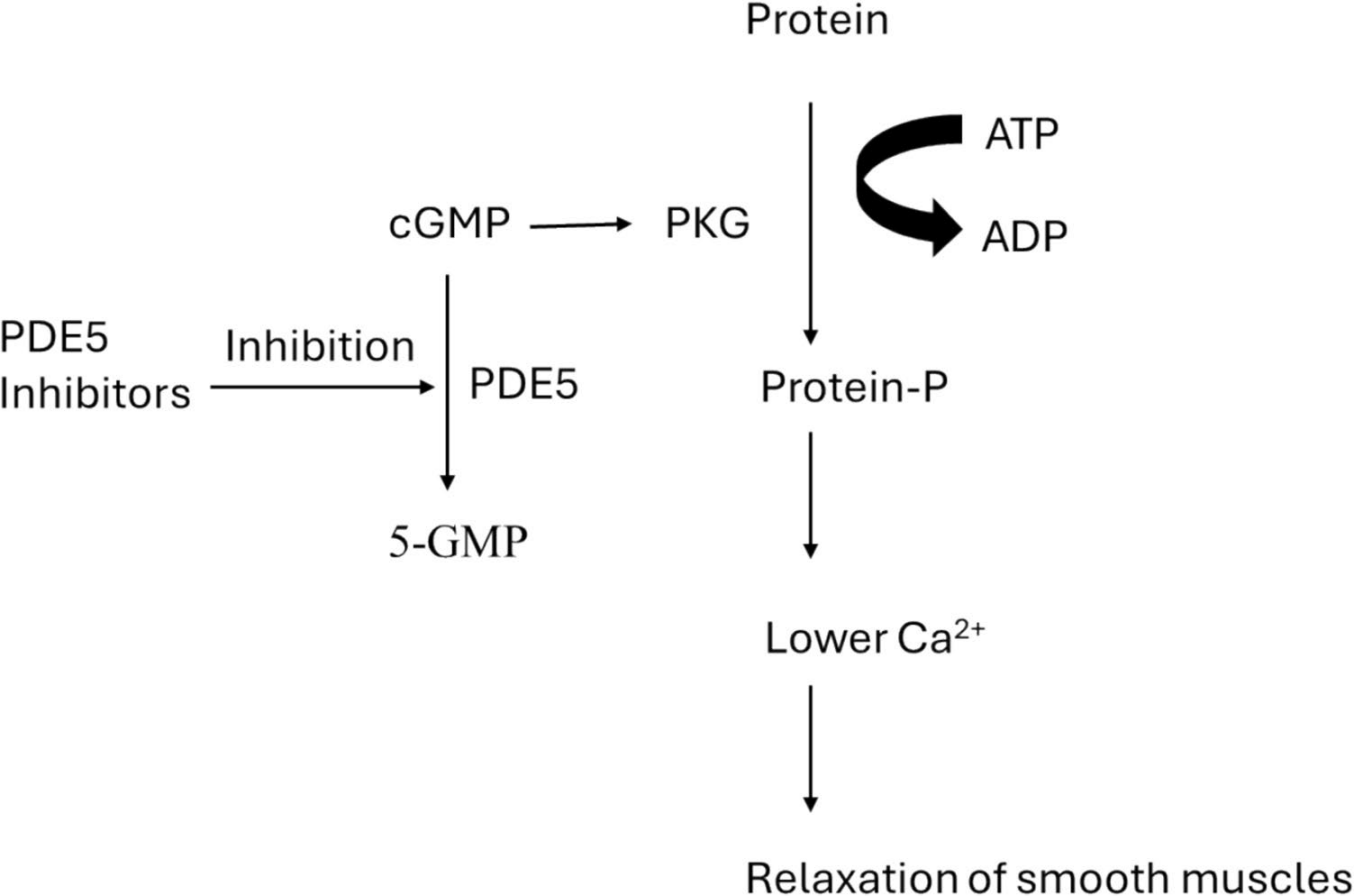
Mechanism of action of PDE5 inhibitors

PDE5 inhibitors is a class of drugs that inhibits PDE5 enzyme, which hydrolyzes cGMP into 5-GMP, leading to the accumulation of cGMP that activates cGMP-dependent protein kinase (PKG). PKG then catalyzes the phosphorylation of several proteins, lowering intracellular calcium (Ca^2+^) levels that results in relaxation of smooth muscles (Fig. 2) [29]. The effect of PDE5 inhibitors on smooth muscles has been employed in a wide variety of pharmaceutical applications, such as treatment of erectile dysfunction (ED), benign prostatic hypertrophy (BPH) and pulmonary arterial hypertension (PAH) [29].

### Classification of PDE5 inhibitors

**Fig. 3:**
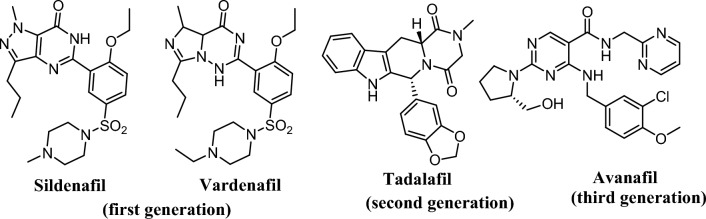
Generations of PDE5 inhibitors

PDE5 inhibitors are classified into three generations: first generation drugs, such as sildenafil and verdenafil, second-generation drugs, such as tadalafil, and third-generation drugs, such as avanafil (Fig. 3).

**Fig. 4:**
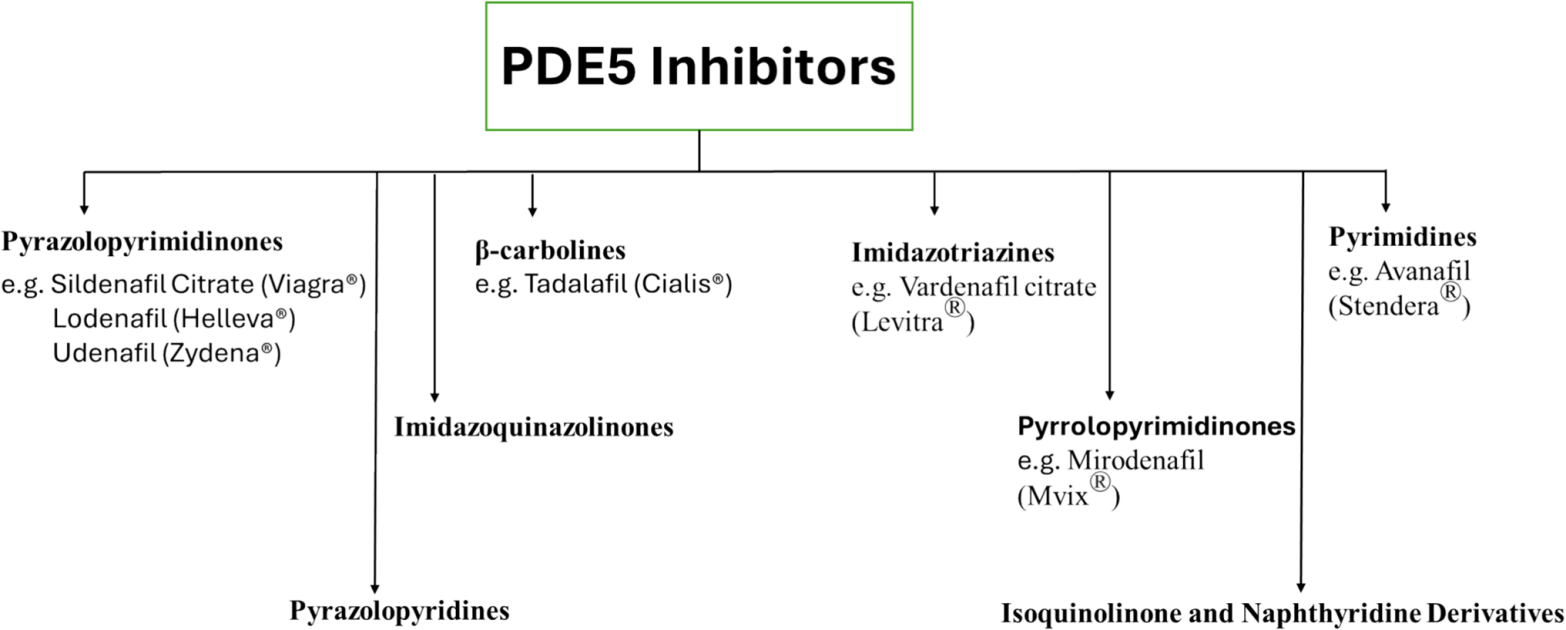
Classification of PDE5 inhibitors with some examples of FDA-approved drugs

**Fig. 5:**
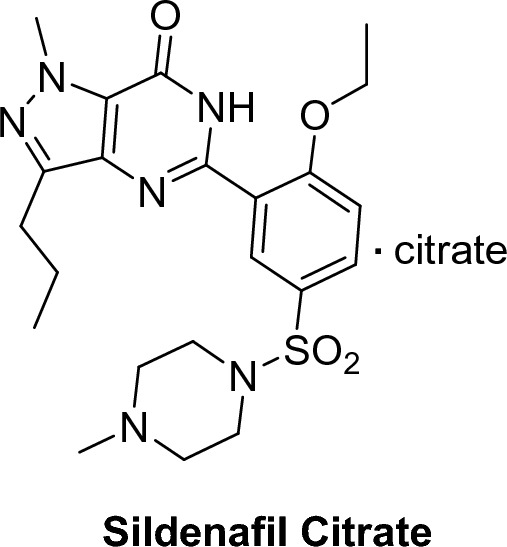
Sildenafil citrate structure

PDE5 inhibitors can also be classified according to their structures into eight categories: Pyrazolopyrimidinones, imidazoquinazolinones, β-carboline, imidazotriazines, pyrrolopyrimidinones, pyrimidines, pyrazolopyridines and isoquinolinone and naphthyridine Derivatives, as shown in Figs. 4 and 5.

#### Pyrazolopyrimidinones [30–32]

##### Sildenafil citrate (viagra®) (First generation PDE5 inhibitor) [30]

Sildenafil citrate, (1-[[3-(6,7-dihydro-1-methyl-7-oxo-3-propyl-1*H*-pyrazolo[4,3-d]pyrimidin-5-yl)-4-ethoxyphenyl]sulfonyl]-4-methyl piperazine), is the first orally potent selective PDE5 inhibitor (IC_50_ = 3.5 nM). In comparison to other known PDEs (tenfold for PDE6, > 80-fold for PDE1, > 700-fold for PDE2, PDE3, PDE4, PDE7, PDE8, PDE9, PDE10, and PDE11), PDE5 is more sensitive to its effects. Sildenafil citrate is almost 4000-fold more selective on PDE5 than PDE3 [30]. However, the non-selective inhibitory effect on PDE3 that is involved in regulation of cardiac contractility, and PDE6 that is present in the retina and is involved in its phototransduction pathway results in several side effects, such as visual disturbances, cardiovascular side effects, in addition to headache and facial flushing [9, 33].

##### Structural features of sildenafil

**Scheme 1.**
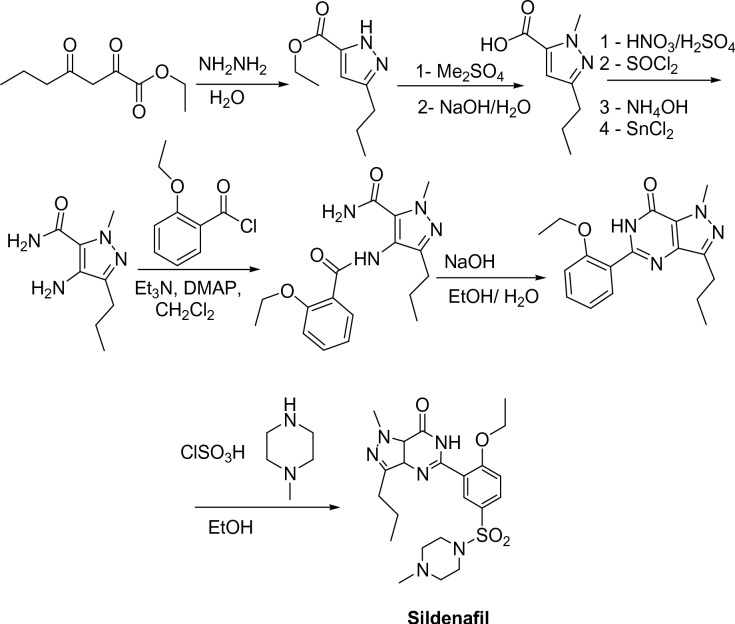
Synthesis of sildenafil

Sildenafil was synthesized through the synthetic pathway as shown in Scheme 1 [34]. The first single X-ray crystal structure investigation of a sildenafil was published in 1999 on iso-sildenafil [34], which varies from sildenafil only by methylation at N2 (instead of N1) of the pyrazolopyrimidine fragment. Despite the widespread attention, it took until year 2005 for sildenafil itself to have its first X-ray diffraction structure determined [35].

The high binding affinity of sildenafil to PDE5 is attributed to certain structural features. For example, pyrzolo[4,3-d]pyrimidine ring forms a hydrophobic interaction with the side chains of the amino acids Val 782, Tyr 612 and phe 820 in the binding pocket. The hydrophobic pocket created by the amino acids Ala 783, Phe 786, Leu 804 and Vl 782 can also fit the ethoxyphenyl group of sildenafil. Moreover, 2’-alkoxy group is an essential group that form a hydrogen bond through its oxygen atom with the pyrimidinone NH, maintaining the coplanarity between the phenyl and heterocyclic systems, in addition to its lipophilicity [36].

##### Quantitative structure–activity relationship (QSAR) of sildenafil

**Fig. 6:**
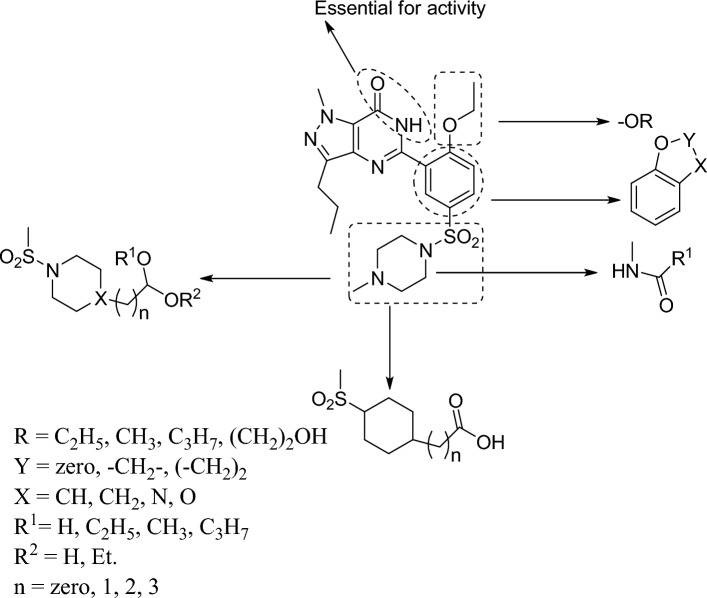
QSAR of sildenafil

**Fig. 7:**
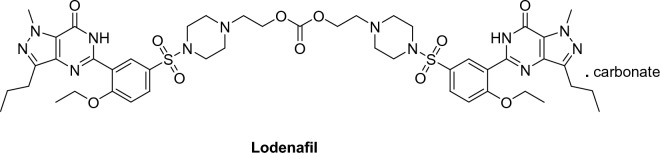
Lodenafil structure

The sturcture activity relationship of sildenafil is shown in Fig. 6. It was reported that replacing methyl group from the pyrazole ring by hydrogen reduced PDE5 activity from (IC_50_ = 27 nM) to (IC_50_ = 82 nM) [33]. It was also observed that introducing polar or charged substituents instead of methyl group of N-methyl piperazine decreased lipophilicity, and thus improved its water solubility [34]. Changing methyl group of *N*- methyl piperazine by p-methoxyphenyl, o-fluorophenyl, p-fluoropenyl, o-chlorophenyl, p- chlorophenyl, p-nitrophenyl or cyclohexyl groups resulted in PDE inhibitors with equal potency to sildenafil [37]. Furthermore, the replacement of *N*-methyl piperazine moiety of sildenafil by carboxylic acid resulted in an increase in the potency to IC_50_ = 0.035 nM, and the inhibitory activity was improved 4 to 38 times as compared to sildenafil [36–38]. Also, the replacement of ethoxy group by hydrogen atom significantly reduced PDE5 affinity by 200-fold. In addition, replacing pyrazole moiety by pyrrole fused to pyrimidine with replacement of the methyl group of piperazine by hydroxyethyl group improved the binding affinity, leading to an enhancement in activity and selectivity [36]. It was also demonstrated that the inhibitory effect and selectivity to PDE5 were reduced by the addition of an ether ring fused to the phenyl moiety in comparison to sildenafil [36]. However, replacing methyl group of *N*-methyl piperazine by tolyl, or *m*-(trifluoromethyl)phenyl groups boosted potency [37]. Finally, the replacement of methyl group at position 1 of pyrazole by ethylpyridyl and propyl group at position 3 by ethyl or methyl groups fostered activity by 343-fold versus PDE6 [36, 39] (Fig. 7).

##### Lodenafil (Hydroxyhomosildenafil) (Helleva®)

**Fig. 8:**
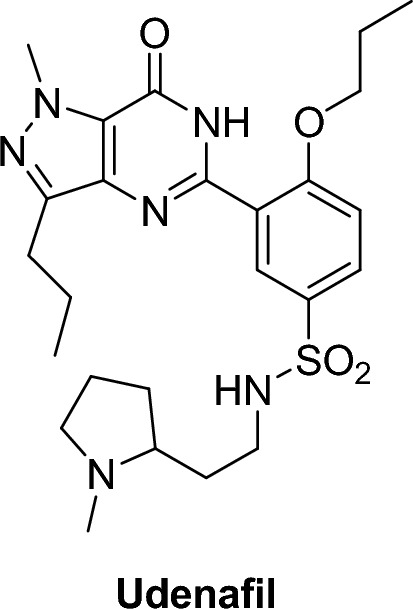
Udenafil structure

Lodenafil, (bis-(2-{4-[4-ethoxy-3-(1-methyl-7-oxo-3-propyl-6,7-di-hydro-1*H*-pyrazolo[4,3-d]pyrimidin-5-yl)-benzenesulfonyl]piperazin-1-yl-ethyl)) carbonate, is a Brazilian-created PDE5 inhibitor that is a dimer made of two sildenafil molecules connected by a carbonate bridge. It acts as a prodrug in which the bridge is hydrolyzed after drug administration, releasing the active ingredient sildenafil [40] (Fig. 8).

##### Udenafil (Zydena®)

Udenafil [41–43], 3-(1-methyl-7-oxo-3-propyl-4,7-dihydro-1*H*-pyrazolo[4,3-d] pyrimidin-5-yl)-N-[2-(1-methylpyrrolidin-2-yl)ethyl]-4 propoxy benzene-sulfonamide, is a strong new PDE5 inhibitor that has been approved in Korea for the treatment of ED. It has a half life (t1/2) (time required for a drug to decrease to its 50% plasma concentration) of 11–13 h and a T max (time required for a drug to reach its maximum concentration Cmax) of 1.0–1.5 h. It is characterized by a relatively rapid onset and a long duration of action [41].

##### Pyrrolopyrimidinones

**Fig. 9:**
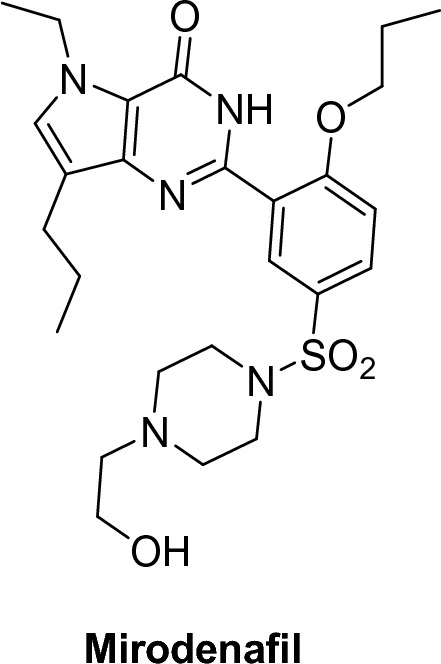
Mirodenafil structure

**Fig. 10:**
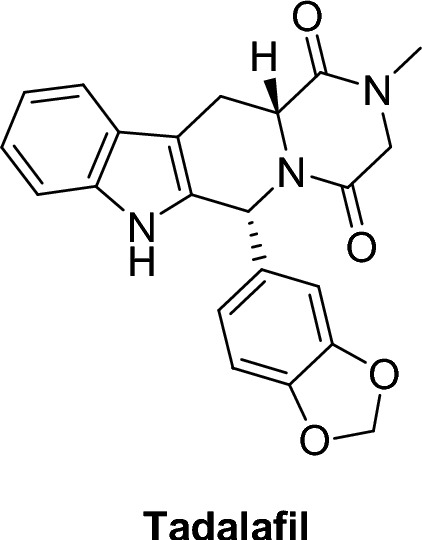
Tadalafil structure

An example of pyrrolopyrimidinones is mirodenafil (Mvix®), (5-ethyl-3,5-dihydro-2-[5-([4-(2-hydroxyethyl)-1-piperazinyl]sulfonyl)-2-propoxyphenyl]-7-propyl-4H-pyrrolo[3,2-d]pyrimidin-4-one). Mirodenafil is a highly effective and selective oral PDE5 inhibitor. It was introduced in Korea in 2007 and was created in 2011 as an oral disintegrating film. According to preclinical research, mirodenafil is 10 × less selective towards PDE5 than sildenafil has a weaker inhibitory effect on other PDEs [42–44] (Figs. 9, 10).

#### β-carboline (second generation PDE5 inhibitor)

##### Tadalafil (Cialis)®

Tadalafil, (6R-trans)-6-(1,3-benzodioxol-5-yl)- 2,3,6,7,12,12a-hexa- hydro-2-methyl-pyrazino [1’, 2’:1,6] pyrido[3,4-b]indole-1,4-dione, is a more potent inhibitor of PDE5 than other phosphodiesterases with IC_50_ of 4 nM. Tadalafil is > 10,000 times more effective inhibitor of PDE5 than PDE1, PDE2, PDE4, and PDE7 enzymes. It is also > 10,000 times more effective in inhibiting PDE5 than PDE3, and 700 times more effective in inhibiting PDE5 than PDE6 [36, 45–48].

##### QSAR of tadalafil

**Fig. 11:**
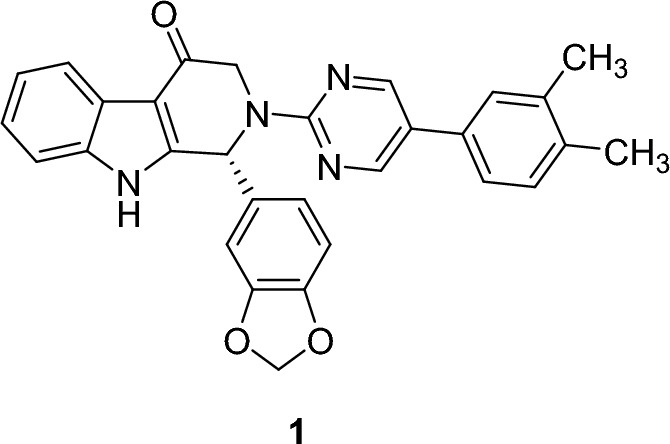
Structure of modified β-carboline

NH group of indole ring is essential for activity; replacing it by the corresponding sulphur containing ring resulted in loss of activity [36]. Also, carbonyl group is essential for activity and removing it resulted in loss of activity [36]. It was reported that replacing pyrazine by other basic rings, such as pyridine or dimethylimidazole raised PDE6 selectivity to 187-fold [36]. When the fused β-carbolines (tadalafil) was modified, a more acidic **1** is yielded, as shown in Fig. 11. the aptitude of NH as a hydrogen bond donor, increases its binding affinity to PDE5 and thus its potency [49].

#### Pyrimidines (third generation PDE5 inhibitor)

##### Avanafil (Stendera®)

**Fig. 12:**
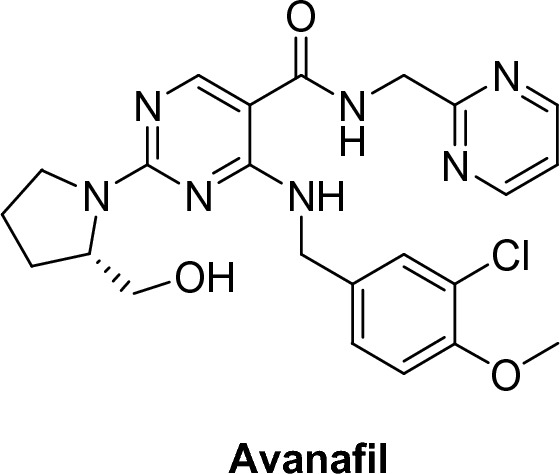
Avanafil structure

**Fig. 13:**
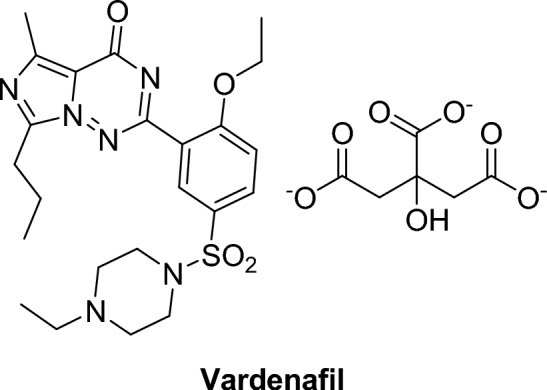
Vardenafil structure

Avanafil, (S)-4-(3-chloro-4-methoxybenzylamino)-2-(2-hydroxymethyl pyrrolidin-1-yl)-N-pyrimidin-2-ylmethyl-5-pyrimidine carboxamide, is a potent inhibitor of PDE5 with IC50 of 5.2 nM. It also has a higher selectivity on PDE5 over PDE6 by 121 fold with less visual disturbance and over PDE1 by 10000 fold with less CVS side effects. It also has a fast onset of action (15–35 min) and t1/2 of less than 0.7–2.5 h [50, 51] (Figs. 12 and 13).

#### Imidazotriazines

##### Vardenafil citrate. (Levitra®)

Vardenafil citrate, 2-(2-ethoxy-5-((4-ethylpiperazin-1-yl) sulfonyl) phenyl)-5-methyl-7-propyl-4a,5-dihydroimidazo[5,1-f][1,2,4]triazin-4(1H)-one, is a more potent and selective PDE5 inhibitor than sildenafil with an IC_50_ of 0.7 nM. It has a rapid onset of action and a prolonged action up to 10 h after oral administration [36, 52].

#### Imidazoquinazolinones

**Fig. 14:**
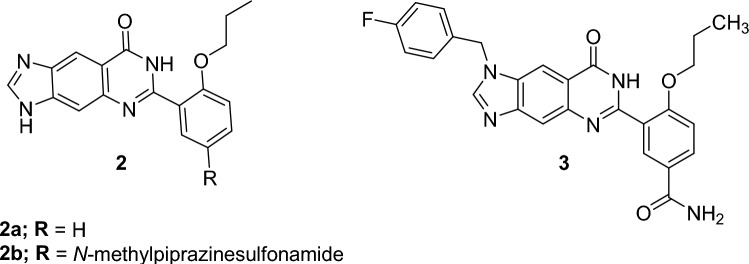
Examples of imidazoquinazolinone-based PDE5 inhibitors

Imidazoquinazolinone derivatives (Fig. 14) were reported as potent PDE5 inhibitors. The potency of **2** was improved tenfold by adding *N*-methylpiperazinesulfonamide, a structural element of sildenafil, to the pendant alkoxybenzene ring [53]. **3** was also reported as a more selective PDE5 inhibitor than sildenafil with an IC_50_ of 0.48 ± 0.1 nM [53].

#### Pyrazolopyridines

**Fig. 15:**
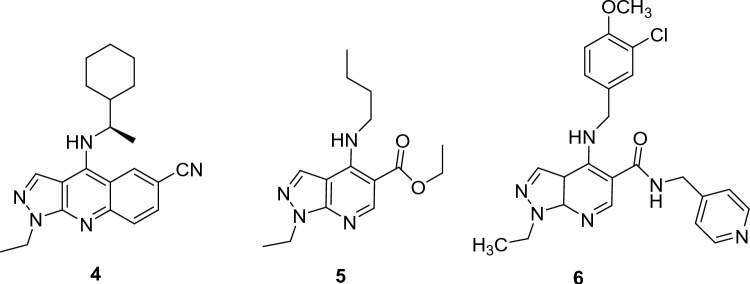
Exmples of pyrazolopyridine-based PDE5 inhibitors

Several pyrazolopyridines were reported as potent PDE5 inhibitors (Fig. 15). **4** was reported as a potent PDE5 inhibitor with IC_50_ of 1.0 nM [54]. Bristol-Myers Squibb researchers selected a number of pyrazolopyridines for PDE5 screening. Through this study, a nonselective PDE5 inhibitor **5** with low potency was discovered (PDE5 IC_50_ = 180 nM) [55]. However, they discovered that **6**, 3-chloro-4-methoxyphenylmethylamine, has the most PDE5 inhibitory efficacy with IC_50_ of 0.8 ± 0.5 nM [55].

#### Isoquinolinone and naphthyridine derivatives

To enhance the potency as PDE5 inhibitors and to improve the isozyme selectivity, a series of 4-aryl-1(2H)-isoquinolinone derivatives were designed, such as **7** and **8**, shown in Fig. 15 [56]. Both compounds are higly selective on PDE5 with high potency (IC50 values are 30 and 21 nM, respectively) [56]. The PDE5 potency and selectivity over PDE6, as well as physicochemical characteristics were improved through the synthesis of 7-picolyloxy derivative (**9)** [57]**.**

**Fig. 16:**
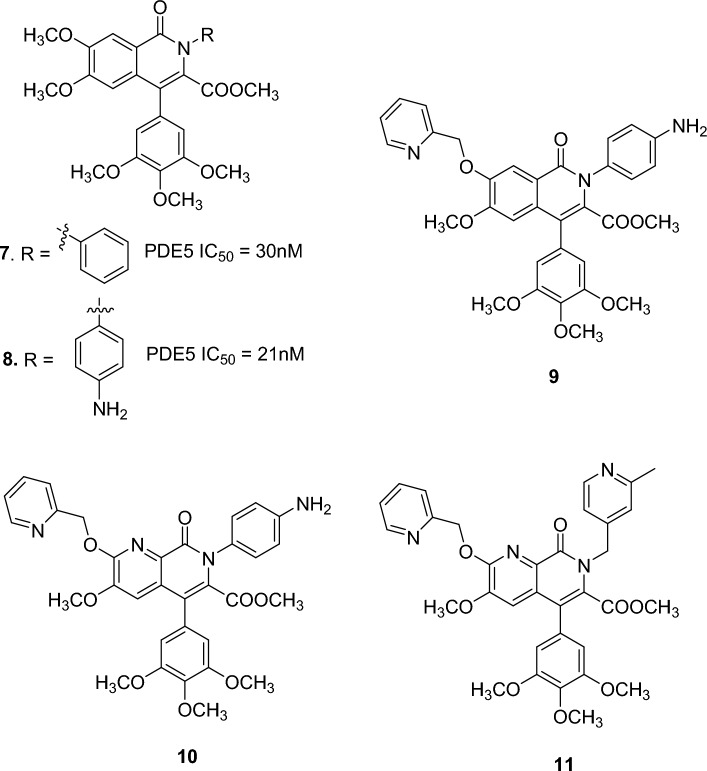
Examples of isoquinolinone and naphthyridine-based PDE5 inhibitors

In order to mimic the *N*7 in cGMP, which contributes to binding at the catalytic site of PDE5, a nitrogen atom was introduced into the isoquinolinone ring, leading to an enhancement of PDE5 inhibitory potency of the resulting 1,7-naphthyridines (**10**) with IC50 of 0.51 nM [58]. Further SAR studies showed that special disposition of basic nitrogen atom at the 7-position was important to exert high PDE5 inhibition and specificity, with optimized methylpicolyl group as in **11** reaching -40-fold selectivity over PDE6 (IC50 = 0.22 nM) [58]. (Fig. 16).

## Therapeutic applications of PDE5 inhibitors

The therapeutic applications of PDE5 inhibitors are summarized in Table 2.

**Table 2:** Therapeutic applications of PDE5 inhibitors

Entry	Class	Therapeutic applications	PDE5 IC50	References
Sildenafil	Pyrazolopyrimidinones	Erectile dysfunction	3.5 nM	[30]
Tadalafil	β-carboline	Erectile dysfunction	4 nM	[36]
Avanafil	Pyrimidine	Erectile dysfunction	5.2 nM	[50]
Vardenafil	Imidazotriazine	Erectile dysfunction	0.7 nM	[52]
3	Imidazoquinazolinones	Erectile dysfunction	0.48 ± 0.1 nM	[53]
5	Pyrazolopyridines	Erectile dysfunction	180 nM	[55]
6	pyrazolopyridines	Erectile dysfunction	0.8 ± 0.5 nM	[55]
7,8	Isoquinolinone	Erectile dysfunction	30 and 21 nM	[56]
10	Naphthyridines	Erectile dysfunction	0.51 nM	[58]
11	Naphthyridines	Erectile dysfunction	0.22 nM	[58]
12	Pyrazolopyrimidineone	Erectile dysfunction	0.8 nM	[62]
13–15	Pyrazolopyrimidineone	Erectile dysfunction	27 nM	[32]
16	Isoquinolinone	Erectile dysfunction	1.50 ± 0.7 nM	[63]
17	Isoquinolinone	Erectile dysfunction	1.69 ± 0.6 nM	[63]
18a-d	Pyrazolopyrimidineone	Antitumor	IC_50_ of 18c = 1.57 nM	[93]
19	thiazolopyrimidine	Antitumor	8.56 ± 0.7µM	[94]
20a-b	9-benzylaminoacridine	Antitumor	7.43 ± 2.69 and 6.29 ± 1.18 µM	[102]
21–23	Thiazole	Anti-inflammatory anti COX I/II	1.00–6.34 µM and 0.09–0.71 µM	[107–111]
24	pyrazolopyrimidinone	Anti-Alzheimer	14.0 nM	[116]
25	pyrazolopyrimidinone	Anti-Alzheimer	11.0 nM	[117]
26	β-carboline	Anti-Alzheimer	3.231 ± 0.327 µM	[119, 120]
27	β-carboline	Anti-Alzheimer	1.530 µM	[119, 120]

### Erectile dysfunction (ED)

Erectile dysfunction (ED) is the continuous inability to obtain or sustain a strong penile erection that allows pleasurable sexual performance [59]. ED is a prevalent illness that affects all age groups, and has a significant negative influence on quality of life. An estimated study mentioned that the prevalence of ED is 39% in 40-year-old men and 67% in those aged 70 years [60]. PDE5 inhibitors, such as sildenafil, vardenafil, tadalafil, and recently avanafil are the primary form of treatment for patients suffering from ED. Those drugs act by inhibiting PDE5 enzyme, which hydrolyzes cGMP to GMP, leading to the accumulation of cGMP in penile tissue (corpus cavernosum) and subsequently causing vasodilatation, and resulting an erection [61].

Several studies have been carried out for the development of novel PDE5 inhibitors for treatment of ED. New pyrazolopyrimidinones were synthesized and their activity on rabbits was compared to sildenafil. It was found out that **12** was as potent as sildenafil, or slightly more efficient, in sustaining the penile erection in the rabbit model. In addition, it has an IC_50_ of 0.8 nM with 20-fold more selectivity on PDE5 against PDE6 [62].
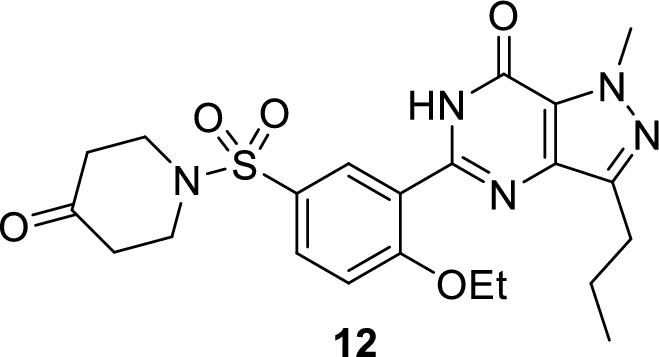


New pyrazolopyrimidinone derivatives **13–15** were developed. It was found out that they exhibited similar PDE5 inhibition effect to sildenafil and highly potent relaxation effect on isolated corpus cavernosum from albino rat model [32].
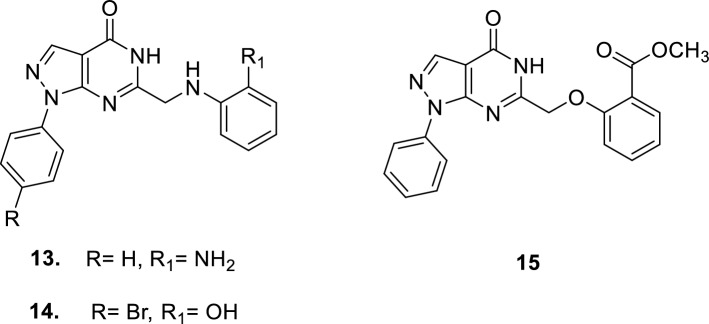


New furoxan coupled spiro-isoquinolino piperidine derivatives **16** and **17** were reported to have PDE5 IC_50_ values of 1.50 ± 0.7 and 1.69 ± 0.6 nM, respectively [63].
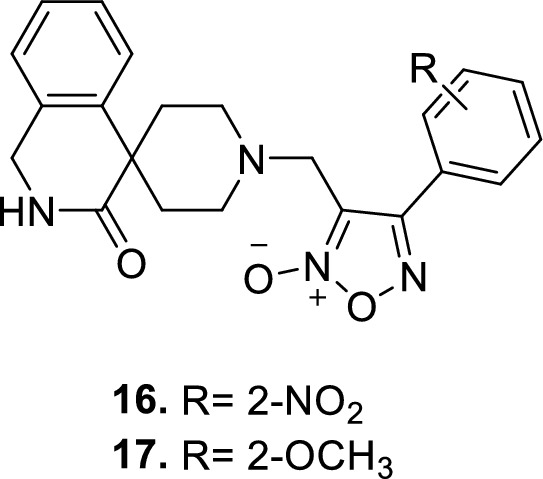


### Benign prostatic hypertrophy (BPH)

BPH is a non-cancerous expansion of the prostate’s size [64]. When sufficiently large, the nodules impinge on the urethra and increase resistance to flow of urine from the bladder. This is commonly referred to as “obstruction”. Tadalafil (Cialis®) has been approved by FDA for the treatment of BPH [65]. Tadalafil, as a PDE5 inhibitor, enhances the Nitric oxide (NO)-cGMP pathway, leading to the relaxation of smooth muscles, inhibition of proliferation of stromal cells and down-regulation of inflammatory responses in the prostate, decreasing prostatic fibrosis [66]. It was demonstrated through clinical trials that combining alpha-1 blockers that are commonly used for the treatment of BPH, such as alfuzosin with PDE5 inhibitors, tadalafil or sildenafil, helps relieve the sexual and urinary symptomps in patients with BPH, or those suffering from both BPH and ED [67]. The efficacy of combining tamsulosin with PDE5 inhibitors in relieving the lower urinary tract symptomos (LUTS) secondary to BPH was also reported [68].

### Eisenmenger’s syndrome

Eisenmenger’s syndrome happens due to the untreated congenital heart defect with intracardiac communication that leads to pulmonary arterial hypertension (PAH), reversal of blood flow, and cyanosis [69, 70]. It is also characterized by ventricular septal defect that leads to shunt from left to right. Thus, the blood contained in left ventricle and aorta is mixed, leading to tissue hypoxia [71, 72]. PDE5 inhibitors, such as sildenafil, tadalafil and vardenafil have been widely used for treatment of PAH by increasing cGMP levels in vascular smooth muscle cells, leading to vascular smootmuscle relaxation, and thus vasodilation [73]. Oral sildenafil (Revatio®) was approved by FDA for treatment of PAH. It exhibits improvement in all pulmonary-function measurements, such as 6-min walking distance (6MWD), pulmonary hemodynamics and survival rates [74].

### Raynaud’s disease

Raynaud’s disease is a condition that is characterized by the ischaemic vasospastic attacks affecting the peripheral artries and arterioles, which leads to peripheral numbness, ulcerations and necrosis [75]. The vasodilating effect of PDE5 inhibitors has been harnessed for treatment of Raynaud’s disease [76]. It was reported that sildenafil and vardenafil reduced the duration and frequency of vasospastic attacks, in addition to improvement of ulcer-healing [77, 78].

### Intrauterine growth restriction (IUGR)

Sildenafil increases uterine blood flow in non-pregnant nulliparous women. Subsequently, it has been suggested as a therapy for intrauterine growth restriction (IUGR) by targeting the uterine vascular bed to decrease vascular impedance and increase uterine blood flow [79]. It was reported that tadalafil treatment improved placental mammalian/mechanistic target of rapamycin (mTOR) signaling in mouse model with L-NG-nitroarginine methyl ester (L-NAME)-induced fetal growth restriction (FGR) and associated preeclampsia (PE). Consequently, it enables fetal growth in FGR mice [80–83].

### Mountain sickness

Mountain sickness is caused by low oxygen levels at high altitudes on mountains that decreases blood-oxygen levels and narrow the pulmonary arteries. Consequently, sildenafil helps alleviate the symptoms of mountain sickness through its vasodilating effect [84].

### COVID-19

PDE5 inhibitors have been recently repurposed for controlling the symptoms of SARS-CoV-2 infections. Life-threatening thromboembolism is one of the complications that COVID patients experienced because of platelet aggregation that is induced through NO/cGMP/PDE5 pathway. Excessive expression of PDE5 in lung tissues also results in interstitial pulmonary fibrosis that is associated with COVID infections [85]. It has been reported that PDE5 inhibitors also inhibit angiotensin-converting enzyme-2 (ACE2), leading to significant pulmonary vasodilation [86]. Also, the accumulation of cGMP in vascular smooth cells and airways caused by PDE5 inhibition results in pulmonary vasodilation, inhibition of vascular hypertrophy and airway relaxation, and hence decreases the risk of lung failure in COVID patients [85, 87].

### Bladder pain syndrome/interstitial cystitis (BPS/IC)

Bladder pain syndrome/interstitial cystitis (BPS/IC) is a chronic disease characterized by chronic bladder pain associated with lower-urinary tract infections [88]. PDE5 inhibitors increase nitric oxide synthase, reduce bladder hyperactivity and improve the microcirculation through its vasodilating effect [88]. Consequently, it was revealed that daily low-dose of tadalafil is well-tolerated, and efficient in treatment for refractory BPS/IC in women [89].

### Kidney diseases

PDE5 inhibitors exert reno-protective effects in patients with diabetic nephropathy or chronic kidney diseases through cGMP-induced vasodilation and improvement renal blood flow [90]. It was also reported that sildenafil reduces albuminuria, glomerular hyperfiltration, glomerular hypertrophy and glomerulosclerosis significantly [90].

### Antiproliferative activity

It was observed that PDE5 expression increases in several types of human cancer cell lines, such as breast cancer (MCF-7), prostate cancer (PC3) and colorectal cancer (HCT16) [91]. It was reported that sildenafil and vardenafil inhibit tumor growth and stimulate caspase-dependent apoptosis of leukemia cells and human colorectal cancer cells [92].

Several PDE5 inhibitors have been also synthesized and tested for their antiproliferative activity. A novel pyrazolo[3,4-d]pyrimidin-4-one derivatives with quinoline scaffold (**18a-d**) was found to have dual potent PDE5 inhibitory activity (e.g. IC_50_ of **18c** = 1.57 nM) and apoptosis induction in several types of cancer cells. **18c** exerts its cytotoxic effect through induction of the intrinsic apoptotic mitochondrial pathways (upregulation of pro-apoptotic protein Bax, active caspase-9 and caspase-3 and decreased levels of antiapoptotic proteins Bcl-2) in HepG2 cells [93].
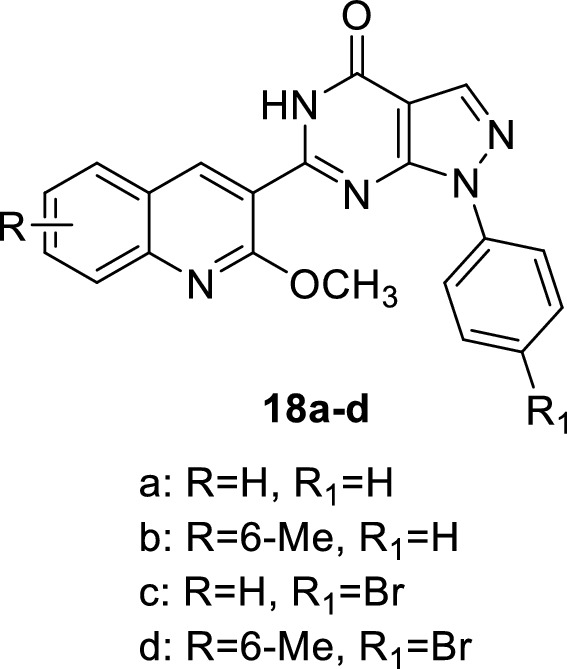


A new thiazolopyrimidine derivative **19** was reported to have a significant PDE5 inhibitory activity (IC_50_ = 0.046 nM) and a strong cytotoxic effect against MCF-7 breast cancer cells with IC_50_ of 8.56 ± 0.7µM [94–101].
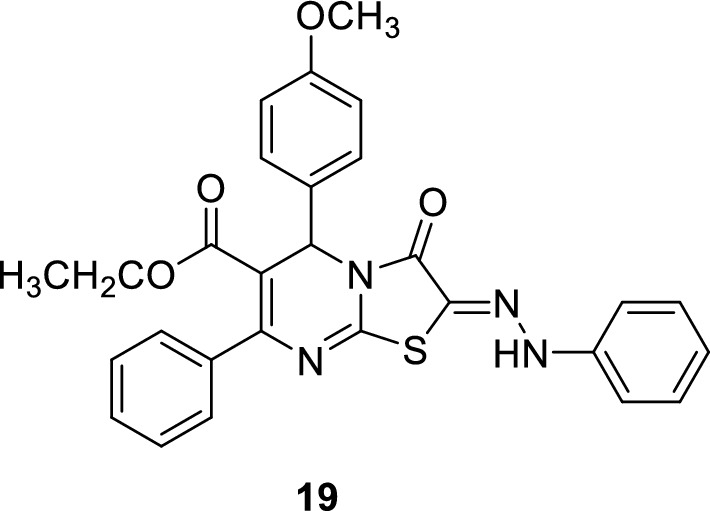


Another novel 9-benzylaminoacridine derivatives **20a-b** were found to inhibit both PDE5 (IC_50_ values are 7.43 ± 2.69 and 6.29 ± 1.18 µM, respectively) and DNA topoisomerase II enzyme, inducing apoptosis of cancer cells. Therefore, **20a-b** show significant cytotoxicity against HCT-116 colorectal adenocarcinoma with IC_50_ values of 7.13 ± 0.24 and 4.84 ± 0.05µM, respectively [102]. The aforementioned findings imply a strong correlation between cytotoxicity and PDE5 inhibition.
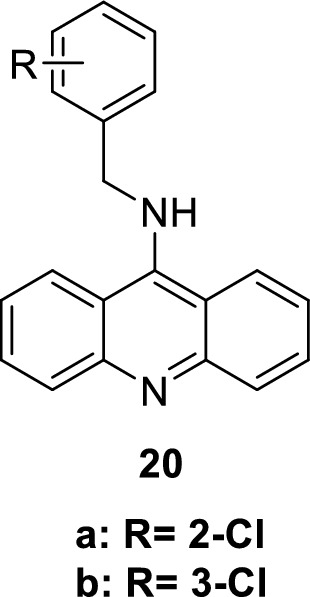


### Treatment of type II diabetes (insulin resistance)

PDE5 inhibitors appear to stabilize insulin resistance in both humans and animals and repair nitric oxide signaling, which is crucial for erectile function [103]. In a recent study, it was found out that glycemic management and erectile function were improved when the glycemic and metabolic consequences of using low dose tadalafil once daily were evaluated for participants with type II diabetes and erectile dysfunction [104]. It was also reported that tadalafil restores the insulin stimulating effect on glucose transport in insulin resistant podocytes [105].

### COX-1/COX-2 inhibitory activity (anti-inflammatory)

PDE5 and COX inhibitory actions have recently been found to be clearly correlated. Sildenafil has been demonstrated to be helpful in the early stages of inflammation in rats with monocrotaline (MCT) induced disease. In addition to its vasodilatation and anti-proliferative actions, sildenafil has direct anti-inflammatory effects, such as preventing pulmonary arterial remodeling and enhancing survival if infected cells [106]. Interestingly, a new family of thiazole scaffolds was recently developed. Hussein et al. reported PDE5 inhibitors **21–23** that exhibit an inhibitory effect on COX-1 and COX-2 enzymes (IC_50_ ranges are 1.00–6.34 µM and 0.09–0.71 µM, respectively) [107–111].
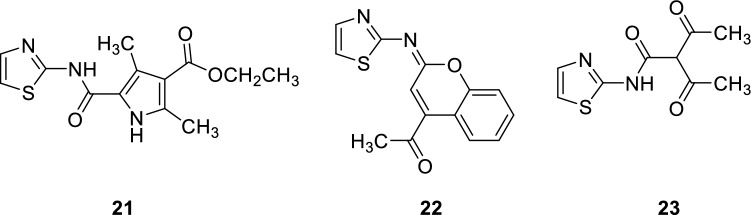


### Anti-Alzheimer

#### Histone deacetylase (HDAC) selective inhibitors

Dual inhibition of histone deacetylase (HDAC) and PDE5 is considered a highly effective strategy for treatment of Alzheimer’s disease (AD) [112]. Inhibition of PDE5 results in accumulation of cGMP that binds to cGMP response element (CREB), stimulating memory-related gene transcription [113]. It was reported that the cognitive decline was reversed and the synaptic plasticity was enhanced in animal models upon treatment with PDE5 inhibitors, such as sildenafil and tadalafil [114, 115]. HDACs is a group of 18 isoforms that are categorized into four classes: I, IIa, IIb and IV. It was reported that inhibiting class I HDACs and HDAC6 that control several memory-related genes restores learning and memory [116, 117]. A novel series of pyrazolopyrimidinone-based dual PDE5 and class I HDAC inhibitors including **24** that has an IC_50_ of 14 and 673 nM for PDE5 and HDAC1, respectively was reported [116]. It was revealed that **24** significantly induced histone acetylation (AcH3K9) and CREB phosphorylation (pCREB) at low concentrations with low cytotoxicity to hepatic cells and neurons after administrating it to Wild type (WT) mouse model of AD (Tg2576) [116]. Moreover, another chemical probe **25** was reported to inhibit both PDE5 and HDAC6 selectively with IC_50_ values of 11 and 15 nM, respectively when evaluated in vivo using the same aforementioned mouse model [117].
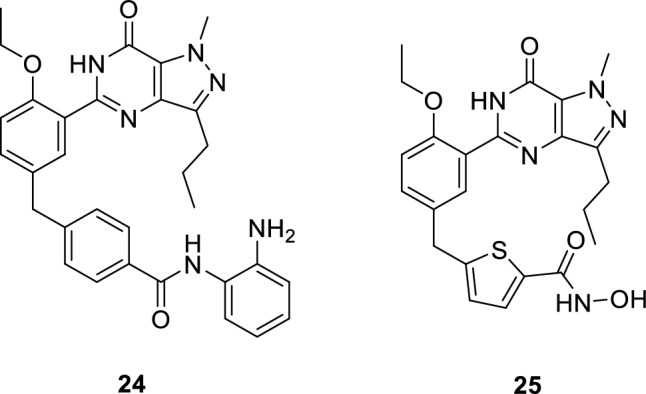


#### Acetylcholinesterase (AChE) inhibitors

Acetylcholinesterase (AChE) has been a potential target for treatment of AD because low levels of acetylcholine that results from over-expressed AChE was revealed in late-stage patients with AD. Consequently, inhibiting AChE increases acetylcholine synaptic levels, and thus mitigates the death of cholinergic nerves [118]. Novel tadalafil derivatives, such as **26** and **27** were reported to effectively reverse the cognitive impairment in mice with scopolamine-induced dementia through their dual inhibitory activity against PDE5 (IC_50_: 3.231 ± 0.327 and 1.530µM, respectively) and AChE (IC_50_ = 0.015 ± 0.003 and 0.032µM, respectively) with adequate blood brain barrier permeability and water solubility [119, 120].
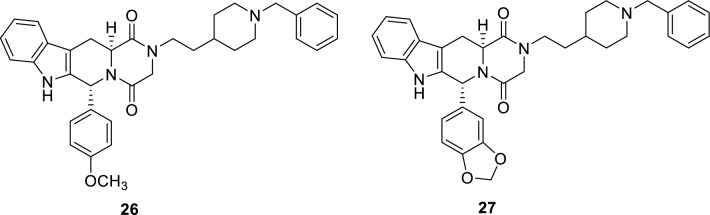


## Recent research patents of PDE5 inhibitors

The diverse therapeutic applications of PDE5 inhibitors motivated the researchers to invent advances in the use of reported compounds or the development of novel scaffolds for new therapeutic applications. For example, one of the recent inventions aimed to combine mirodenafil with CORT-108297, a glucocorticoid receptor (GR) antagonist that is used for treatment of post-traumatic stress disorder (PTSD), for treatment of dementia. That combination exerts its effect by inhibiting β-secretase and thus reducing the levels of amyloid beta protein (Αβ) that accumulates in the nerve cells of AD patients and causes neuronal death [121]. In another study, **28** was used for treatment of anterior ischemic optic neuropathy (AION), which is characterized by dysfunctional optic nerve and retina leading to significant loss of visual acuity. It stimulates the release of NO, enhancing the ocular blood flow and the visual acuity [122].
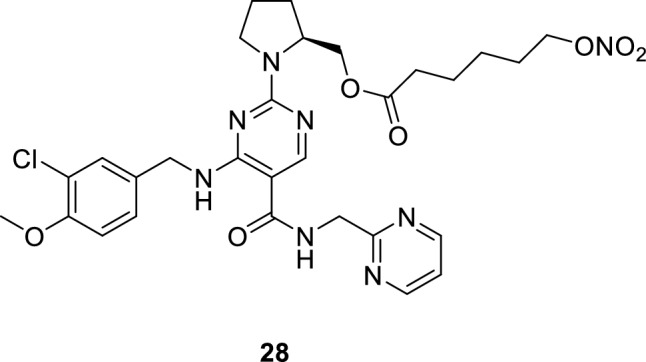


Recent studies also showed that hemi-licorice isoflavone B (**29**) and echinatin (**30**) exert a strong inhibitory activity on PDE5 and has an outstanding potential for treatment of ED [123, 124].
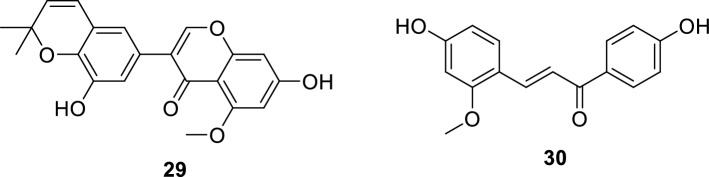


It was also reported that low-dose sildenafil when combined with mepivacaine, a local anaesthetic, in addition to glucose reduces ATP-evoked intracellular signaling, decreases intracellular calcium signaling in chondrocytes and upregulates growth factors that are included in the generation of cartilage. Consequently, that combined therapy can be utilized in the treatment of osteoarthritis (OA) [125]. In another study, sildenafil can be delivered directly to the muscles of the pelvic floor through intravaginal apparatus to increase blood flow and induce muscle anabolism for the treatment of female urinary incontinence (UI) [126].

One of the inventions also showed that ellagic acid (**31**) exerts PDE5 inhibitory effect that can be utilized for treatment of ED and prostatic hypertrophy [127]. A novel antitumor PDE5 inhibitor (**32**) was also reported for treatment of prostate cancer [128].
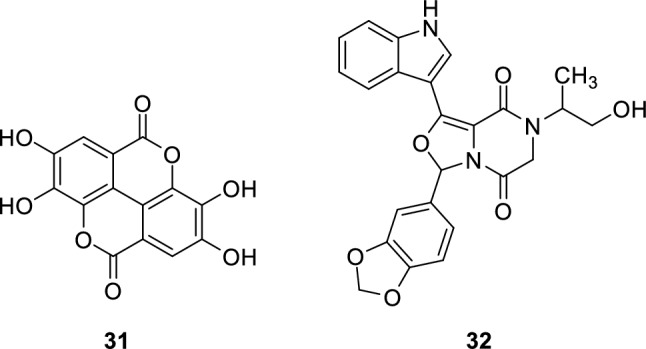


A new arylpyrazolopyrimidinone derivative (**33**) was reported to exert a dual PDE5 and androgen receptor (AR) inhibitory activity that showed a significant cytotoxic effect on breast cancer cell lines [129].
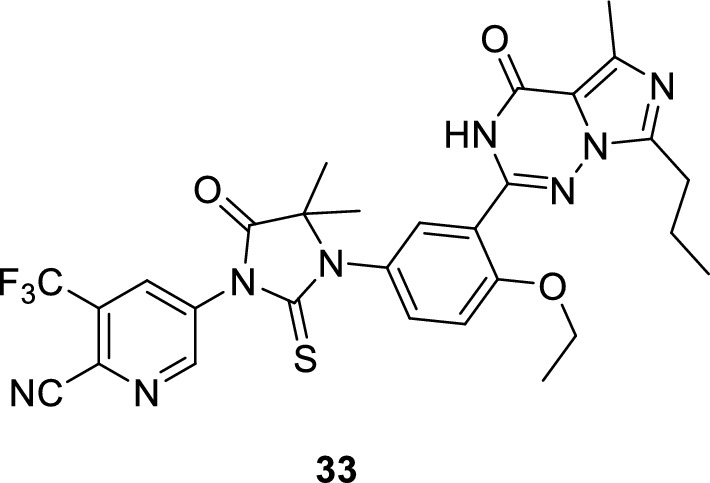


Another invention pertains to PDE5 inhibitor, such as sildenafil that is used for treatment of Maternally Inherited Leigh syndrome (MILS), a neuropathy that is associated with mitochondrial Complex V deficiency, by reducing the mitochondrial membrane potential [130].

## PDE5 inhibitors in clinical trials

There are some PDE5 inhibitors that have been in clinical trials for a different therapeutic application. For example, the effectiveness of tadalafil has been evaluated in clinical trials for treatment of preeclampsia (hypertensive disorder of pregnancy) [131]. It is also evaluated for management of chronic prostatitis/chronic pelvic pain syndrome: a randomized double-blind placebo controlled clinical trials [132]. The safety and pharmacokinetic behavior of youkenafil, an oral PDE5 inhibitor for treatment of ED, has been evaluated in phase I trials that are conducted on 24 individuals (12 elderly and 12 young patients) upon oral administration of 100mg single dose [133]. Aildenafil citrate has been also evaluated for treatment of ED though phase I clinical trials on 20 patients after multiple oral administration of 30mg and 60 mg doses [134]. Examples of PDE5 inhibitors in clinical trials are shown in Table 3.

**Table 3:** PDE5 inhibitors in clinical trials

Drug	Therapeutic application	Clinical trial phase	Reference
Tadalafil	Preeclampsia	Phase II	[131]
Tadalafil	Chronic prostatitis/chronic pelvic pain syndrome	Phase II	[132]
Youkenafil	Erectile dysfunction	Phase I	[133]
Aildenafil	Erectile dysfunction	Phase I	[134]
Simmerafil	Erectile dysfunction	Phase II	[135]

## Pharmacokinetics and pharmacogenetics of PDE5 inhibitors

Sildenafil has specific pharmacokinetic properties including: (1) it is administered orally; (2) it is absorbed fast with 3–4 h half-life (the shortest among PDE5 inhibitors); (3) its steady state distribution volume equals 105L, suggesting that the medication is distributed into the tissues; (4) it has a strong affinity to plasma proteins; (5) its oral bioavailability is 40%, indicating that it is metabolized through first pass metabolism by hepatic CYP3A4/5, CYP3A5, CYP2D6, and CYP2C19 [136–138]. It is suggested that its pharmacodynamic heterogeneity between people accounts for both therapeutic efficacy and side effects as there is no correlation between pharmacokinetic parameters and pharmacological effectiveness. Age and concurrent use of CYP3A4/5 inhibitors, such as ketoconazole, cimetidine, erythromycin, antacids, and HIV protease inhibitors, can boost drug levels in plasma. However, taking CYP3A inducers such rifampin, phenobarbital, phenytoin, and carbamazepine will result in lower plasma levels of sildenafil [137].

## Contraindication of PDE5 inhibitors

PDE5 inhibitors have been utilized for a wide variety of pharmaceutical applications. Nontheless, they are contraindicated in case of specific health conditions, such as recent stroke or heart attack, severe heart disease, such as unstable angina or irregular heartbeat (arrhythmia(, severe heart failure, uncontrolled high blood pressure, uncontrolled diabetes and low blood pressure [138]. They are also contraindicated with administration of nitrates [4, 33].

It was found out that sildenafil, vardenafil and tadalafil cause a reduction in blood pressure. Consequently, they are contraindicated in hypotensive patients. The concomitant use of PDE5 inhibitors and α-adrenoreceptor antagonists should be also prevented to mitigate the risk of hypotension [139, 140]. In addition, it was reported that vardenafil causes prolongation of heart rate corrected QT interval (QTc), resulting in irregular heart rhythm. As a result, vardenafil should not be co-administered with antiarrhythmic drugs, such as procainamide, quinidine, amiodarone and sotalol [141].

PDES inhibitors should also be used with caution and under medical supervision by patients with severe renal impairment and hepatic cirrhosis because of decreased drug clearance from the blood, leading to increased exposure to the drug effects. Furthermore, increased exposure to sildenafil may cause harmful effects, such as hypotension or priapism (prolonged penile erection that may end with irreversible tissue damage) [139].

## Side effects of PDE5 inhibitors

The side effects of PDE5 inhibitors are attributed to non-selective inhibition of other PDE enzymes. Those side effects include headache, visual disturbances, facial flushing, and back pain [142–144]. There have been a small number of case reports of seizures and myocardial infarction with PDE5 inhibitors administration [145].

## Conclusion

In addition to their effect on ED, PDE5 inhibitors are crucial for other pharmaceutical applications. Several FDA-approved PDE5 inhibitors have been employed in treatment of different health conditions, such as type II diabetes (insulin resistance), mountain sickness, Eisenmenger’s syndrome, Raynaud’s disease, intrauterine growth retardation (IUGR), benign prostatic hypertrophy (BPH) and Bladder pain syndrome/interstitial cystitis (BPS/IC). Moreover, the researchers have successfully developed novel PDE5 inhibitors that exert significant inhibitory activity on PDE5 enzyme, and thus potential anti-Alzheimer, anti-proliferative, and COX-1/COX-2 inhibitory effects (anti-inflammatory). The safety of administration of PDE5 inhibitors into human body should be assessed by evaluating their pharmacokinetic behavior. PDE5 inhibitors are safe to be orally administered as they are appropriately cleared from the blood circulation and metabolized by the liver through first-pass metabolism. However, they should not be used by patients with liver diseases, high blood pressure, arrythmia and uncontrolled diabetes and renal impairment. In a response to high demand for PDE5 inhibitors in the medical field, researchers have been repurposing existing PDE5 inhibitors and developing new structurally related scaffolds for more effective incorporation of PDE5 inhibitors into medical practice, especially in case of recent global health concerns, such as COVID-19. The researchers have been also working on increasing the selectivity of inhibitors on PDE5 than other PDEs to overcome their side effects.

## Data Availability

No datasets were generated or analysed during the current study.
